# L'endocardite à Bartonella en Tunisie: particularités lésionnelles et évolutives

**DOI:** 10.11604/pamj.2013.16.24.1262

**Published:** 2013-09-23

**Authors:** Rania Hammami, Dorra Abid, Leila Abid, Abir Znazen, Mourad Hentati, Adnene Hammami, Samir Kammoun

**Affiliations:** 1 Service de Cardiologie, Hopital Hédi Chaker, Sfax,Tunisie; 2 Laboratoire de Bactériologie, Hopital Habib Bourguiba, Sfax, Tunisie

**Keywords:** Bartonella, endocardite, chirurgie, mortalité, Bartonella, endocarditis, surgery, mortality

## Abstract

L'endocardite à Bartonalla est une infection ubiquitaire, son diagnostic est difficile vu qu'il s'agit souvent d'endocardite à hémoculture négative. Le but de cette étude est d'analyser les particularités lésionnelles et évolutives de cette entité dans un pays du nord d'Afrique, la Tunisie et de démontrer la gravité de cette infection. Nous avons étudié rétrospectivement les dossiers médicaux de 20 patients atteints d'endocardite à Bartonella, confirmée selon les critères de Dukes modifiés. L’âge moyen de nos patients était 37 ans avec une prédominance masculine (SR=3). Tous nos malades avaient un niveau socio-économique bas. Le motif essentiel de consultation était la dyspnée, 6 patients étaient admis dans un tableau d'insuffisance cardiaque congestive. Une prédilection des lésions au niveau de la valve aortique a été notée (14 cas). Quatorze patients avaient des végétations endocarditiques avec une taille qui dépasse 10 mm chez 8 malades. La majorité des patients (18 patients) présentaient une régurgitation valvulaire massive en rapport principalement avec des mutilations importantes (6 cas de ruptures de cordages mitraux, 2 cas de déchirures des sigmoïdes aortiques, un cas de perforation valvulaire aortique, un cas de désinsertion de prothèse mitrale). Quinze malades (3/4) avaient nécessité une chirurgie à la phase active de la maladie, l'indication majeure était l'insuffisance cardiaque. Une complication neurologique était notée chez 2 malades et une complication rénale chez 3 malades. Treize patients étaient guéris, 5 malades étaient décédés et 2 malades opérés ont présenté une réinfection à staphylococcus aureus et à candida albicans en postopératoire. L'endocardite à Bartonella est une infection grave. Cette Bactérie possède un potentiel destructif important. Le recours à la chirurgie est quasi constant. La morbi-mortalité est élevée. La recherche de cette bactérie devrait être alors systématique chez nos malades suspects d'endocardite d'autant plus que la bartonellose est endémique sur nos terres.

## Introduction

Depuis l'avènement des nouvelles techniques de diagnostic microbiologique, on suggère que les bactéries de type Bartonella sont incriminées dans 3% de l'ensemble des endocardites infectieuses en Europe [1] et jusqu’à 13% dans l'Afrique du nord [2, 3]. Il s'agit d'une endocardite subaigüe à hémoculture négative, le diagnostic est généralement tardif, le traitement est encore controversé et l’évolution est grevée d'une morbi-mortalité importante. Le but de cette étude est de rapporter les caractéristiques lésionnelles et évolutives de l'atteinte endocarditique à Bartonella et de démontrer la gravité de cette infection dans un pays du Nord d'Afrique, la Tunisie.

## Méthodes

Il s'agit d'une étude rétrospective établie dans le service de Cardiologie de l'Hôpital Hédi Chaker entre Janvier 1997 et Juillet 2010 en collaboration avec le laboratoire de Bactériologie de Sfax. Cette étude a inclut tous les cas d'endocardites infectieuses (EI) à Bartonella définies comme certaines ou possibles selon les critères de Dukes modifiés[4].

Sur le plan microbiologique, le diagnostic de Bartonella est retenu soit en obtenant une sérologie positive anti-bartonella avec un titre élevé ≥ 1/800 par la méthode d'immunofluorescence, soit en isolant le germe au niveau des cultures de sang, sérum ou valve, soit en utilisant les nouvelles techniques de biologie moléculaire (la réaction de polymérisation en chaine de l'ADN: PCR).

Nous avons relevé à partir des dossiers médicaux des malades les éléments suivants: les données démographiques (âge et sexe) et épidémiologiques (contact avec les chats, alcoolisme, absence de domicile fixe, niveau socio-économique); les données cliniques (fièvre > 38°, altération de l’état général, souffle cardiaque, signes cutanés et articulaires); les données échocardiographiques (siège et nombre de l'atteinte valvulaire, taille et mobilité des végétations, abcès, anévrysme, rupture ou perforation de valve); les formes compliquées: complications infectieuses, neurologiques, rénales et hémodynamiques; la stratégie thérapeutique: le traitement antibitiotique, le recours ou non à la chirurgie; l’évolution: guérison, rechute (définie selon les nouvelles recommandations de l'endocardite infectieuse [5] comme une infection au même germe dans les premiers 6 mois suivant le premier épisode), réinfection (infection par le même germe au delà de 6 mois ou par un germe différent quelque soit le délai [5]) ou décès.

## Résultats

Durant la période de l’étude, 225 cas d'EI certaines ou possibles ont été diagnostiqués dans notre centre, les bactéries de type Bartonella ont été identifiées comme le germe infectant chez 20 patients soit une prévalence de 8,9%.

### Données épidémiologiques et cliniques

L’âge moyen de nos patients était de 37,71±9.2 ans avec des extrêmes allant de 19 à 58 ans, une prédominance masculine a été notée (SR = 3). Aucun de nos patient n’était alcoolique ni sans domicile fixe. Par contre, tous les patients avaient un niveau socio-économique bas; un contact avec les animaux a été rapporté chez 13 malades puisqu'ils vivaient dans un endroit rural mais sans notion d'infection aux poux du corps. Deux patients était immunodéprimés, le premier sous corticoïdes au long cours pour un lupus systématique et le deuxième était trisomique 21, connu porteur d'une leucémie aigue. La dyspnée était le motif de consultation le plus fréquent et elle a concerné 13 patients (65%). Six malades étaient admis dans un tableau d'insuffisance cardiaque congestive. L'examen cardiaque retrouvait un souffle cardiaque chez tous les patients, et une fièvre > 38° chez les 2/3 des malades.

Toutes les HC étaient négatives. Les sérologies à Bartonella étaient positives chez 17 patients. L'analyse des valves cardiaques par PCR a été réalisée uniquement chez 5 patients parmi les 15 opérés. Elle était positive à Bartonella chez 4 patients. Par la méthode de Western Blot, nous avons identifié 17 infections à *Bartonella quinta* et 3 infections à *Bartonella Hanselea* (Tableau 1)

**Tableau 1:** Caractéristiques épidémiologiques, cliniques et microbiologiques de l'endocardite à Bartonalla en Tunisie

**Données démographiques et épidémiologiques**	
Age (ans)	37,71±9.2 (extrêmes: 19 à 58)
Sexe ratio	3 (15Hommes/5Femmes)
**Facteurs prédisposant:**	
Alcoolisme	aucun patient
Sans domicile fixe	aucun patient
Niveaux socio-économique bas	tous les malades
Milieu rural	13 patients/20
Contact avec les animaux (chat, chien)	13 patients/20
Infection par les poux du corps	aucun patient
Immunodéprimé	2 patients/20
Antécédents de valvulopathies	11 patients/20
Porteur de prothèse valvulaire	1 patient/20
**Données cliniques**	
Fièvre > 38°	13 patients/20
Altération de l’état général	6 patients/20
Dyspnée	13 patients/20
Souffle cardiaque	Tous les patients
Insuffisance cardiaque congestive	6 patients/20
Signes cutanées	1 patient/20
**Données bactériologiques**	
Espèce incriminée	*B quinta*: 16 patients, *B Hanselea*: 4 patients
Hémocultures négatives	Tous les malades
Sérologie positive à un titre > 1/800	17 patients/20
PCR (+)	4 patients/20

### Données échocardiographiques

Tous les patients ont bénéficié d'une échocardiographie trans-thoracique (ETT) et seulement 13 patients ont été examinés par échocardiographie trans-‘sophagienne (ETO). L'apport diagnostic de l'ETO était bénéfique chez 2 malades et a concerné essentiellement des anomalies en faveur de l'EI qui n’étaient pas objectivées à l'ETT (végétation mitro-aortique

L'infection a concerné la valve cardiaque native chez 19 patients et une prothèse mitrale mécanique chez un seul patient. La localisation aortique était prédominante (14 patients) avec une atteinte mitro-aortique chez 3 malades. Aucun cas d'endocardite sur c'ur droit n'a été diagnostiqué. Les végétations et les mutilations valvulaires sont les lésions élémentaires les plus fréquemment rencontrées dans notre série. La présence de végétations était notée dans 14 cas avec une taille> à 10mm chez 8 patients. Les mutilations valvulaires concernaient essentiellement l'appareil mitral. Une rupture de cordage était notée chez 6 patients (33%), une déchirure des sigmoïdes aortiques a été observée dans 2 cas et une perforation aortique dans un cas. Dans notre série, nous n'avons objectivé aucun cas d'abcès annulaire que ce soit à l'ETT ou à l'ETO. Cependant un abcès annulaire a été constaté en péri-opératoire chez un seul patient. La majorité des sujets (18 patients) présentaient des régurgitations importantes, une hypertension artérielle pulmonaire importante > 50mmHg a été rapportée chez 13 malades à l'admission.

Le ventricule gauche était hypercontractile témoignant d'une insuffisance valvulaire aigue chez 9 patients et de fonction systolique conservée chez 7 malades. Quatre patients avaient une dysfonction systolique avec une hypokinésie globale, deux sujets avaient paradoxalement une régurgitation mitrale grade II et des végétations ne dépassant les 10 mm, alors que les 2 autres avaient une valvulopathie importante (Tableau 2).

**Tableau 2:** Données échocardiographiques de l'endocardite à Bartonella en Tunisie

**Localisation**	Aortique: 11 patients
	Mitrale: 5 patients
	Mitro-aortique: 3 patients
	Prothèse mitrale: 1 patient
	Prothèse aortique: aucun patient
	Cæur droit: aucun patient
	Matériel endocavitaire: aucun patient
**Végétations**	Nombre de malades ayant des végétations: 14 patients/20
	Taille >10mm: 8 patients
	Taille >15 mm: 3 patients
**Mutilations valvulaires**	Rupture de Cordage de la valve mitrale: 6 patients/20
	Déchirures sigmoïdiennes aortiques: 2 patients/20
	Perforations sigmoïdiennes: 1 patient/20
	Désinsertion de prothèse: 1 patient/20
**Abcès**	Annulaire: aucun patient
	Valvulaire: 1 patient/20
**Régurgitations valvulaires**	Insuffisance aortique grade III-IV: 15patients/20
	Insuffisance mitrale grade III-IV: 12 patients/20
	Insuffisance aortique grade I-II: aucun patient
	Insuffisance mitrale grade I-II: 2 patients/20
**Fonction du ventricule gauche**	Hypercontractile (FEVG > 70%): 9 patients
	Conservée (FeVG: 45-70%): 7 patients
	Abaissée (FeVG < 45%): 4 patients

### Données thérapeutiques et évolutives

Un traitement antibiotique a été instauré chez tous les malades à part deux patients décédés le jour de l'admission, avant même de commencer l'antibiothérapie. Le traitement antibiotique consistait en une association d'ampicilline ou pénicilline G avec la gentamicine dans 14 cas et une association de la céftriaxone avec la gentamicine chez 4 cas, vu un contexte probable d'allergie à la pénicilline. La durée de traitement était aux alentours de 6-7 semaines. Une chirurgie urgente a été indiquée chez 11 malades vu un tableau de d'insuffisance cardiaque à l'admission ou au cours des premières jours de l'hospitalisation mais n’était réalisée que chez 8 malades, chez les 3 autres, l’évolution était très rapide; 2 patients évoluaient en choc réfractaire et décédés le jour de l'admission et le 3^ème^ présentait un accident vasculaire hémorragique par rupture d'anévrysme cérébrale, condition entravant la chirurgie, l’évolution était également fatale chez ce dernier. Parmi les 9 patients traités médicalement, 7 malades ont nécessité une chirurgie tardive (après 2 semaines d'antibiothérapie) vu la persistance de végétations de grandes tailles compliqués déjà d'accident vasculaire ischémique cérébral chez un patient. Durant l’évolution, 3 malades ont développé une insuffisance rénale aigue, une biopsie rénale réalisé chez 2 patients avait conclut à une glomérulonéphrite extra-capillaire infectieuse. Cette insuffisance rénale était réversible chez tous les patients.

L’évolution était fatale chez 5 patients: l'insuffisance cardiaque était la cause de décès chez 4 patients (2 décédés à l'admission, un décédé 4 jours après la chirurgie à cause d'une désinsertion de prothèse, un décédé 5 mois plutard à cause d'une insuffisance cardiaque terminale) alors que l'hémorragie cérébrale était la cause de la mort chez le 5^ème^ patient. Chez le reste des sujets, l’évolution était favorable cher 13 patients avec guérison complète, une réinfection postopératoire a été notée chez 2 patients, à staphylocoque aureus chez l'un et à candida albicans chez l'autre (Tableau 3).

**Tableau 3:** Evolution de l'endocardite à Bartonella

**Complications**	Insuffisance cardiaque: 11 patients/20
	Neurologique: 2 patients (1 AVC ischémique, 1 AVC hémorragique)
	Rénale: 3 patients/20
Guérison	13 patients
Décès	5 patients/20
Rechute	Aucun patient
Réinfection	2 patients/20

## Discussion

La bartonelle est une bactérie à gram négatif, identifiée la première fois comme l'agent de la fièvre de Tranchée en 1909, durant la première guerre mondiale [6]. Cette bactérie existe sous forme de plusieurs espèces, seulement une dizaine d'espèces sont pathogènes pour l'homme et sont responsables de plusieurs infections comme la maladie de griffe de chat, l'angiomatose bacillaire, la réto-rétinite, les méningites, les hépatites, les bactériémies sévères. L'atteinte du cæur peut concerner les 3 tuniques, mais l'atteinte la plus commune est l'endocardite infectieuse, celle-ci est décrite pour la première fois en 1993[7], soit après huit décades de la primo-infection de l'Homme par cette bactérie; les espèces responsables de cette infection sont essentiellement B quintana, B henselae et B elizabethae.

L'endocardite à Bartonella est une maladie ubiquitaire, sa prévalence est différente selon les régions, elle varie entre 1 et 3% de l'ensemble des EI et 28% de l'ensemble des EI à hémocultures négatives [1, 8] dans les pays occidentaux alors qu'elle est plus fréquente dans les pays en voie de développement représentant jusqu’à 13% de l'ensemble des EI dans l'Algérie [2] et 8,9% dans notre série, indiquant ainsi un aspect endémique de cette infection dans ces régions.

L'absence de domicile fixe et l'alcoolisme identifiés comme les principaux facteurs prédisposant dans la publication de Raoult et al [8], ne semblent pas être des facteurs épidémiologiques déterminants chez nos malades; la principale caractéristique épidémiologique dans notre série était le niveau socio-économique très bas. Cette maladie semble infecter aussi bien les sujets immunodéprimés essentiellement les malades atteint de VIH que les sujets immunocompétents [9]. Dans notre série, 2 patients seulement étaient immunodéprimés ce qui prouve que la gravité de l'atteinte n'est pas liée en particulier au terrain sous-jacent.

Sur le plan clinique, l'endocardite à Bartonella se présente habituellement sous forme de tableau subaigu [8] avec des symptômes non spécifiques incluant la fièvre, la fatigue, l'altération de l’état général’ Dans notre série, l'insuffisance cardiaque semble le motif le plus fréquent de découverte de la maladie, signant d'emblée la gravité de la maladie. Sur le plan lésionnel, les deux tiers des EI à Bartonella touchent la valve aortique [10], cette prédilection infectieuse est encore non expliquée. Certaines publications [10, 11] concernant une localisation multivalvulaire ont été rapportées, il s'agit le plus souvent d'endocardite mitro-aortique ou aortico-tricuspide. L'atteinte isolée du c'ur droit semble exceptionnelle [12].

L'endocardite sur prothèse est aussi une forme rare, caractérisée par des lésions de désinsertions, de perforations et de défaillance cardiaque [10, 11]. Dans la revue de Raoult [8], concernant 101 patients atteints d'endocardites à Bartonella, un seul patient était porteur de prothèse mécanique, comme c’était le cas dans notre série. Les végétations de grandes tailles et les mutilations valvulaires compliquées de régurgitations massives sont fréquentes au cours des endocardites à Bartonella. Dans une série de Lepedi et al [13], les auteurs ont comparé les particularités histopathologiques entre 15 patients atteints d'EI à Bartonella et 25 patients atteints d'EI non à Bartonella et ont prouvé que cette espèce à un potentiel destructif très important vis-à-vis des valves cardiaques; elle est responsable d'inflammation à cellule mononucléaire, d'une fibrose extensive, de larges calcifications et d'une néo-vascularisation peu développée témoignant ainsi d'une inflammation chronique. Les auteurs indiquent par contre que les végétations sont généralement non de grandes tailles comparant aux endocardites non à Bartonella. Paradoxalement, Raoult et al [10] confirment que cette infection est souvent associée à des végétations larges, détectables dans 90% des cas en ETT. Dans notre série, les 2/3 des malades avaient des végétations dont la majorité de grande taille. La caractéristique lésionnelle majeure chez nos malades était les fuites valvulaires importantes quasi constantes. Les lésions de type abcès valvulaire ou annulaire n’était pas fréquentes, observées chez un seul malade, ce résultat est comparable à la série de Raoult et al [10], parmi 101cas d'EI à Bartonella, un seul cas d'abcès valvulaire a été décrit et deux cas de déchirure de valve.

Dans la littérature, les complications des EI à Bartonella sont peu discutées même dans les plus séries les plus larges, des cas isolés ont été publiés concernant des complications neurologiques [14, 15], ou hémodynamiques [11]. Dans la série algérienne [2], parmi 14 patients atteints d'EI confirmées, il y avait 3 cas d'insuffisance cardiaque, 2 cas de péricardite, un cas d'insuffisance rénale et 2 cas d'embolie vasculaire, soit la moitié des patients ayant présenté une EI compliquée. Dans notre série, la complication la plus fréquente était l'insuffisance cardiaque congestive présente chez plus que la moitié des malades à l'admission ou au cours de l’évolution. Cette complication était l'indication principale de chirurgie et la première cause de décès. En effet, elle peut être expliquée par l'effet des mutilations valvulaires importantes responsables des régurgitations massives ainsi que la grande taille des végétations mais aussi probablement par une composante myocarditique secondaire à Bartonella, on avait noté d'ailleurs une dysfonction du ventricule gauche avec une hypokinésie globale chez 4 malades dont deux avaient une fuite mitrale moyenne et des végétations non très larges. Motriciol et al [11] a rapporté un cas de myocardite fatale chez un adulte immunocompétent atteint d'une endocardite à Bartonella et a prouvé que cette myocardiopathie est secondaire à ce germe. Les complications neurologiques et rénales étaient mois fréquentes chez nos malades et ceci coïncident avec les données des différentes publications [2, 8, 10].

Le traitement optimal des endocardites à bartonella est non encore élucidé vu l'absence de larges séries, dans les dernières recommandations de la société Européenne de Cardiologie [5], on propose l'association d'Ampicilline ou Céftriaxone ou doxycycline avec la gentamicine mais aucune étude n'a prouvé la supériorité d'un schéma thérapeutique par rapport à l'autre. De toute façon, le traitement médical seul ne semble pas suffir, plus que 90% des patients [10] nécessitent une chirurgie, en particulier les formes à *Bartonella quinta*. Chez nos malades, parmi les 5 malades non opérés, 3 étaient décédés, alors que 2 malades seulement décèdent parmi 15 patients opérés, ceci prouve bien la nécessité de recours à la chirurgie au cours de cette infection.

Malgré qu'il s'agit majoritairement d'endocardite sur valve native, l'EI à Bartonella était considérée par Raoult et al [8, 10] comme associée à une mortalité élevée allant jusqu’ à 31% malgré le traitement médico-chirurgical, les causes de décès étant essentiellement l'insuffisance cardiaque réfractaire et la défaillance multi-viscérale. Dans la série de Benslimani et al [2], le taux de mortalité était 21%, dans notre série était 25%. Cette surmortalité a été expliquée [10] par la survenue de l'infection chez des sujets alcooliques, à bas niveaux socio-économiques d'une part et par le retard et la difficulté du diagnostic d'autre part. Une autre cause pouvant être suggérée à partir de notre série, c'est l'agressivité de Bartonella vis-à-vis des valves cardiaques.

## Conclusion

L'endocardite à Bartonella est une infection grave responsable d'une morbi-mortalité accrue. Sa survenue chez des sujets ayant un niveau socio-économique bas, la difficulté diagnostic, le potentiel destructif et mutilant de la bactérie, le recours quasi constant à la chirurgie et l'absence de consensus concernant le traitement antibiotique sont tous des facteurs qui peuvent expliquer la gravité de cette maladie. Etant donné la distribution endémique des Bartonelloses et avec l'amélioration des moyens de diagnostic microbiologique dans notre pays, la recherche de cette bactérie chez tous nos patients suspects d'EI devrait être systématique.
